# The General Medical Council

**Published:** 1894-12-15

**Authors:** 


### THE GENERAL MEDICAL COUNCIL.

The autumn session of the General Medical Council, which commenced on November 27th, was this year unusually prolonged, mainly in consequence of the number of penal cases which were ready for adjudication. With reference to these, a new mode of action, which was determined upon at the May meeting, came for the first time into operation; and by means of it the Council is now able to postpone a final decision, and to adjourn a case either *sine die* or to some future session. The defendant is thus afforded a *locus pœnitentiæ*, and is placed somewhat in the position of an accused person who is ordered to come up for judgment if called upon. On the further hearing of the case the Council may receive additional evidence, either in favour of the accused person or against him. The Council had recourse to the new procedure in two instances; finding that Dr. Brecknell had committed the offence of covering an unqualified person named Kirby, and adjourning the further consideration of his case *sine die*; and finding that the same offence had been committed by Mr. Thomas Richards in the case of one Owen, and ordering Mr. Richards to attend at the November session of next year, and to produce evidence as to his conduct in the interval. The name of Lewis Lamb Bailes, who had been convicted of felony, was ordered to be erased from the Medical Register on the ground of such conviction; and the erasure of the following names was ordered after each case had been heard and considered by the Council: Herbert Tibbits, Robert Masters Theobald, Daniel Costelloe, Charles Oakes, and John Alexander Brown. A charge of covering an unqualified person was brought against John Patrick O'Connell, but, in the opinion of the Council, was not sustained. In three of the foregoing cases the charge against the accused persons was not of covering, but of aiding and abetting quackery; the persons so aided being the notorious "Count Mattei" by Theobald, the "Electric Battery Company" by Tibbits, and a self-styled "hernia specialist," one Sherman, by Oakes.

A question of considerable importance which came before the Council was that of the certificates of competency given to midwives by various private societies, institutions, or individuals. After full discussion, and after the inspection of some of the certificates, the Council passed the following resolutions:—

(*a*) That the Council, being of opinion that certain documents issued by various societies or persons as diplomas of education and examination in midwifery are "colourable imitations" of diplomas conferring a legal right to admission to the Medical Register, and both contravene the spirit of the Medical Acts and are calculated to deceive the public, hereby give notice that from the present date the issue of such "colourable imitations" by registered practitioners will be regarded as conduct infamous in a professional respect.

(*b*) That, in the opinion of the Council, the form of the certificate now before the Council, purporting to be granted by the Obstetrical Society of London on July 20th, 1894, is such that it may be regarded as a document coming within the purview of the foregoing resolution, and that this opinion be communicated to the President of the Council of the Obstetrical Society.

The Council received a report from its Pharmacopœia Committee with regard to the steps which are being taken for the compilation of a new edition of the work; and, after recommitting the report for the insertion of fuller details, adopted it when again presented. Professor Attfield has been appointed editor of the new edition, and it is proposed to extend its authority from Great Britain and Ireland to India and the Colonies. For this purpose communications have been opened with the chief representatives of medicine and pharmacy in all the British dependencies.

The Examinations Committee presented reports from the inspector and visitors upon all the final examinations for medical diplomas in the United Kingdom, and announced that a report discussing the various questions raised in them, would be prepared by the committee in readiness for the May meeting of the Council. The full consideration of the examinations in question was therefore adjourned, and the same was done with reference to the examinations of the various licensing bodies for degrees or diplomas in "Public Health." With regard to these, the Council possesses full statutory power to register them or to refuse to register them; and it has laid down the general principle that registration will only be granted to those which may be taken to imply a distinctively high proficiency, both scientific and practical, in each and all of the branches of knowledge which enter into the subject.

The session terminated on Thursday, December 6th.

